# Interannual Variability of Fisheries Economic Returns and Energy Ratios Is Mostly Explained by Gear Type

**DOI:** 10.1371/journal.pone.0070165

**Published:** 2013-07-29

**Authors:** Verena M. Trenkel, Fabienne Daurès, Marie-Joëlle Rochet, Pascal Lorance

**Affiliations:** 1 Institut français de recherche pour l'exploitation de la mer, Ecologie et modèles pour l'halieutique, Nantes, France; 2 Institut français de recherche pour l'exploitation de la mer, UMR Aménagement des usages des ressources et des espaces marins et littoraux, Plouzané, France; Technical University of Denmark, Denmark

## Abstract

According to portfolio theory applied to fisheries management, economic returns are stabilised by harvesting in a portfolio stocks of species whose returns are negatively correlated and for which the portfolio economic return variance is smaller than the sum of stock specific return variances. Also, variability is expected to decrease with portfolio width. Using a range of indicators, these predictions were tested for the French fishing fleets in the Bay of Biscay (Northeast Atlantic) during the period 2001–2009. For this, vessels were grouped into eight fishing fleets based on the gears used and exploited species were grouped into five functional groups. The portfolio width of fleets ranged from 1–3 functional groups, or 4–19 species. Economic fleet returns (sale revenues minus fishing costs) varied strongly between years; the interannual variability was independent of portfolio width (species or functional groups). Energy ratio expressed by the ratio between fuel energy used for fishing and energy contained in landings varied from 0.3 for purse seines to 9.7 for trawlers using bottom trawls alone or in combination with pelagic trawls independent of portfolio width. Interannual variability in total sale revenues was larger than the sum of species specific sales revenue variability, except for fleets using hooks and pelagic trawlers; it increased with the number of species exploited. In conclusion, the interannual variability of economic returns or energy ratios of French fisheries in the Bay of Biscay did not decrease with the number of species or functional groups exploited, though it varied between fleets.

## Introduction

Fisheries are confronted by variability in resources and markets generating income risks. To guard against environmental risks, Costanza et al. [1] advocated the application of the principles of financial portfolio management to environmental management. A portfolio strategy consists in asset diversification and is likely to be advantageous when different assets change in different ways over time. The likelihood of this to happen should increase with portfolio diversity or width. Translated into the fisheries context, a portfolio strategy requires a diversity of fishing gears, fishing areas or target species at the individual fisher level [2], [3]. Fisheries management can encourage diversification of activities [4], [5]. Edwards et al. [6] went a step further and proposed a portfolio approach to fisheries management which explicitly considers the trade-offs between harvesting functional groups, i.e. species connected via the food web, in such a way that the harvesting modifies the ecosystem to a desired profitable state.

The economic returns from fishery catches are the total revenues (also called landed value or turnover), minus the extraction costs. If the landings are made up of several species, then, the variance of the composite return is the sum of the species return variances plus the covariance of these returns. Thus, if returns from the different species are negatively correlated, the portfolio return variance is smaller than the sum of species specific return variances; the difference between the two variances should increase with the number of species in the portfolio. Negative correlation between economic returns of species in the portfolio is a necessary condition for a portfolio strategy to be advantageous, that is, less risky by providing more stable returns, compared to a single target strategy. This means that, assuming that extraction costs are similar for different species, revenues of species in a portfolio need to be negatively correlated. Total revenue is the result of quantity landed and ex-vessel fish price (price fetched by fishers per kg landed fish or shellfish). Quantity landed in turn is the result of resource availability and fishing tactics. The conditions that can make covariances negative – that is, portfolio strategies advantageous – are (i) ecological interactions resulting in negative correlations between species abundances, (ii) negative correlations between ex-vessel fish prices of different species, or (iii) appropriate fishing tactics. By contrast, portfolio strategies may not be worth developing when (1) species abundances fluctuate in a synchronized way, for example in response to environmental conditions, (2) ex-vessel fish prices are positively correlated, or (3) non-selective fishing gears limit the fishers' ability to take a targeted catch. Besides, negative correlations between quantity landed and ex-vessel fish price due to price flexibility (assuming exogenous supply - inverse demand system) might stabilize returns from single species fisheries and make portfolio strategies less attractive. Negative ecological interactions include predation and competition. Competition within a functional group can result in compensation thus negative correlations between those species. By contrast, predation is more likely to link species belonging to different functional groups – or the functional groups themselves. On the economic side, due to global trading, fish price indices (taking account of price and quantity) of different seafood products have become positively correlated across the globe [7] and across countries within Europe [8], a tendency which could work against a portfolio effect. The relationship between quantity landed and ex-vessel fish price can be both positive and negative depending on the species, as observed with the São Paulo wholesale market [9]. Here, we investigate whether the portfolios of French fleets in the Bay of Biscay demonstrate the expected positive relationship between portfolio width and increased stability.

The Bay of Biscay (Northeast Atlantic) is a diverse ecosystem offering a range of fishing opportunities for fleets using a diversity of fishing gears [10]. Its long fishing history has lead to the collapse of more than one species, such as blackspot seabream (*Pagellus bogaraveo*) in the 1960s and 70s [11], entailing changes in the species composition [12] and in landings [13]. Similarly, fishing methods have evolved over time as vessel sizes, legislation and fuel prices changed. In the early 2000s, the number of trawlers decreased more strongly than those of vessels using passive fishing gears, most likely a result of increased fuel prices [10].

The currently available resource in the Bay of Biscay is the outcome of historical interactions between ecosystem dynamics and past exploitation. Both functional group biomass and species biomass within functional groups have changed over the last three decades [14]. In addition, landings also depend on market demand and are determined by fishing tactics, i.e. fishing areas, seasons and gears used, and regulations, including available landings quotas. The current fleet structure, in particular the distribution of vessel sizes, is the result of fishing capacity building in the 1950s–1980s [15] followed by European vessel decommissioning schemes of recent decades which aimed at reducing capacity [16] but also a consequence of national fiscal policy [17].

In this study we evaluate returns from the fisheries in the Bay of Biscay by French fleets and their variability. Vessel membership to a fleet is defined by the gear (or combination of gears) used (Table 1). The fleet level averages out individual vessel differences and allows us to concentrate on the main patterns. We consider returns created by landings both in monetary (€) and energetic value (kJ). Food energy supplies are important for world food security [18]. Energy content in fresh fish is linearly related to lipid content and positively related to protein content [19]. Thus, energetic values of landed fish also inform on protein supplies derived from seafood. Further, by working with energy as one of the units, we can compare extracted energy to the fuel energy used for the extraction. One of the current challenges of fisheries is to supply marine products with reduced fossil fuel consumption and thus reduced greenhouse gas emissions [20].

**Table 1:** Average fleet size (N), number of functional groups and species landed, variance ratio of functional groups and species and annual economic return (landed value - costs) and energy ratio (fuel energy/landings energy) for the French fleets fishing in the Bay of Biscay averaged over the period 2001–2009; minimum and maximum annual values in brackets.

Fleet	Gears$	N	No. functional groups	No. species	Variance ratio functional groups*	Variance ratio species*	Economic return € per vessel (min; max)	Energy ratio € J^−1^ per vessel (min; max)
Mixed trawlers	otter, midwater, pelagic trawls	493	3	19	1.9	2.9	25400 (13300; 37300)	9.7 (8.2; 11.0)
Pelagic trawlers	pelagic trawls	33	2	7	0.3	0.4	38100 (100; 77900)	4.7 (2.6; 7.0)
Dredgers	dredges	90	3	14	2	2.6	7000 (3600; 10600)	7.1 (5.1; 9.3)
Hooks	set longlines, handlines	88	1	4	0.9	0.9	13600 (8100; 18400)	4.2 (3.2; 5.0)
Netters	trammel nets, driftnets, set gillnets	182	2	9	1.3	1.9	40600 (28400; 60100)	5.9 (5.1; 6.5)
Potters	pots,	29	1	4	1	1.1	2000 (−3500; 40900)	3.9 (2.5; 6.0)
Purse seiners	purse seines	23	1	7	1.3	1.4	22200 (−24800; 83200)	0.3 (0.2; 0.4)
Several gears	active & passive gears	497	3	15	1	1.6	6100 (1900; 12100)	8.1 (7; 9.3)

* Variance ratio is interannual variance of fleet total revenues (per vessel) divided by the sum of interannual variances of fleet functional groups or species revenues (per vessel).

$ Only dominant gears are listed.

Portfolio width can be measured in several ways. Kasperski and Holland [21] described vessel portfolio width by the spread of revenue across species using the Simpson diversity index and related it to the interannual variability of revenues over a 30 year period. The positive relationship was robust to an alternative measure of portfolio width, the number of fisheries a vessel had participated in. This interpreted to mean that relationships, if they existed, should be robust to the way portfolio width is measured. Here we measure portfolio width at two levels: (i) at the species level – the traditional level of stock management, which makes sense from an economic point of view, since prices are species-specific and (ii) at the functional group level, which has been proposed as a management unit in an ecosystem-based perspective of fisheries management [22]. At the species level, we expect compensation between species to favour the portfolio strategy. The functional group level makes sense from a fishing tactic point of view, since in a given gear a mix of species from a functional group may constitute the entire catch and in the case of profound ecosystem changes trophic cascades between functions groups can occur [4] – fishers might adapt by diversifying functional groups.

The results of this study indicate no reduction in interannual economic return variability with increasing portfolio width. The differences found between fleets point towards the importance of portfolio composition in species and functional groups.

## Materials and Methods

### Data

#### Landings and economic data

Annual landings data (weight and value) by species or commercial species group (several species sold together) from French vessels operating primarily in the Bay of Biscay (ICES Divisions VIIIa & VIIIb) during the period 2001 to 2009 were extracted from the Ifremer Fisheries Information System database [23]. Vessel technical characteristics (length, power, etc.) and fishing operation data were available from the same database.

Economic data were only available for a sample of vessels and collected using questionnaires [24]. Therefore, total annual fishing costs consisting of total fixed and variable operational costs were estimated for all vessels using the generalised additive models (GAM) developed in Daurès et al. [25]. Daurès et al. fitted models for annual labour costs and for all other operational costs (including fuel costs) as a function of vessel technical characteristics and fishing operation data. Annual fuel consumption estimates were derived from the separate Daurès et al. model for fuel costs by dividing estimated fuel costs by the average *fuel price* per litre of each year.

To transform *fuel consumption* (l) to *fuel energy* (kJ), it was assumed that a litre of fuel contained 38307.66 kJ (derived from information in Wiviott and Mathews [26]). Note that no fuel consumption estimates were available for 2000 and 2001.

#### Species data

All species were assigned to one of six functional groups (see species lists in Supplementary Material). The energy content of landings by fleet was calculated by multiplying landings by a species-specific energy content value (kJ per g wet weight) taken from Spitz et al. [19] who analysed samples collected in autumn in the Bay of Biscay. No information was found for some species in which case values from taxonomically close species were used (Table S1). Though lipid content is known to vary seasonally and by sex [27], [28], these variations were assumed to be smaller than inter-specific differences and hence ignored.

### Analysis

#### Fleet-food web interactions

**Figure 1:**
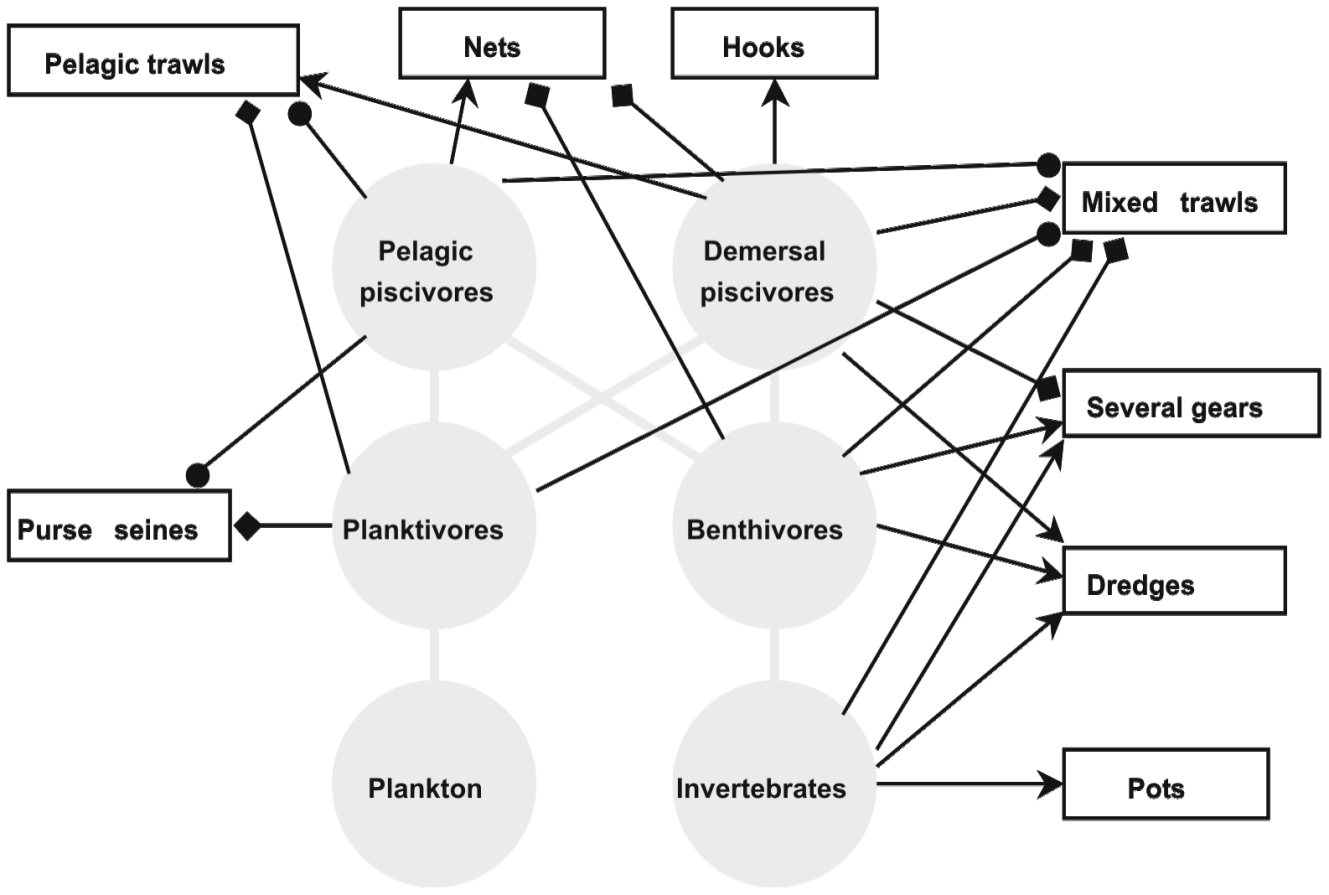
Interactions between major French fishing fleets and functional groups in the Bay of Biscay (2000–2009). Round arrows: >10% of functional group landings energy (kJ) is caught by the fleet; pointed arrows: functional group represents >10% of fleet landings value (€); square arrows: functional group represents >10% in fleet landings value and >10% of functional group landings energy is caught by the fleet. Trophic interactions are depicted by grey lines.

To characterize the major interactions between fishing fleets and functional groups a schematic food web model was drawn which retained the main features of the Bay of Biscay system [29]: six broad functional groups organised into a pelagic and a demersal food chain and eight fishing fleets (Figure 1). The links drawn among functional groups were based on diet information [29]. They allow to visualize how the effects of fishing different functional groups might propagate through the food web and thus shape it. To characterize the interaction between functional group *i* and fishing fleet *j*, the *contribution C*_ij_ of fleet *j* to the landings *L*_ij_ (in kJ) of functional group *i* and the *dependence D*_ij_ of fleet *j* on functional group *i* in terms of revenue *Q*_ij_ (in Euros) were calculated:
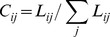


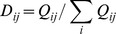



#### Fleet returns

Three variables were used to measure annual returns from fishing functional groups for each fleet: *economic return*, *energy ratio* and *value-per-energy-extracted*. The economic return *Rj* of fleet *j* was calculated by subtracting total annual fleet operational costs *O_j_* from total annual revenues *Q_j_*:
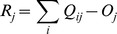
The *energy ratio E*_j_ is simply the total fuel energy *G*_j_ consumed by the fleet in a given year divided by the sum of the energy contained in the species landings:
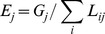
To compare the energetic efficiency among fleets, the sales value-per-energy-extracted *V*_ij_ (€ kJ^−1^) was calculated per fleet and functional group:
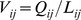
Note that monetary variables were not corrected for inflation as the study period was rather short.

#### Testing the portfolio theory

To evaluate the predictions of portfolio theory, the ratio between the interannual variance of annual revenues (€) and the sum of interannual species or functional group revenue variances was calculated for each fleet. According to portfolio theory, this ratio should be smaller than one. Further, the negative correlation between the interannual variation of economic returns and the number of species or the number of functional groups was tested using Spearman's rank correlation test (one-sided test). The same approach was used for the interannual variation of energy ratios. Under portfolio theory, negative relationships are expected as wider portfolios should dampen temporal fluctuations.

The number of species contributing 90% of total revenues during the study period was taken as the first measure of portfolio width. For this species were ordered by their contribution from maximum to minimum. To determine the number of functional groups exploited by a fleet, only functional groups contributing on average to at least 10% of annual total revenues were counted.

To investigate the relationship between species ex-vessel fish prices within functional groups, time series of scaled (normalised) average unit prices were plotted.

## Results

### Fleet-food web interactions

**Figure 2:**
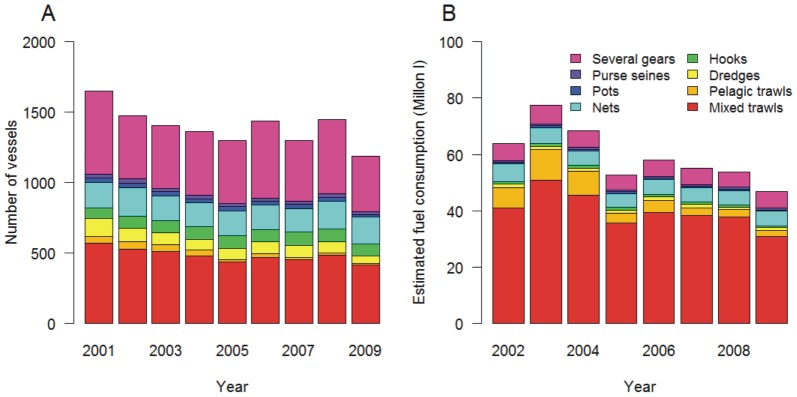
Number of vessels in French Bay of Biscay fleets (A) and estimated annual fuel consumption (l) by fleet (B). No fuel consumption estimates were available for 2001.

The number of vessels decreased over the study period in most fleets (Figure 2a). The fleets using mixed trawls and several gears were by far the biggest fleets, each with nearly 500 vessels on average, while the smallest fleets were those using pelagic trawls, purse seines and pots consisting of around 30 vessels each (Table 1). The estimated annual fuel consumption by fleet followed the same decreasing pattern as the number of vessels with mixed trawlers being the most important fuel consumers (Figure 2b).

**Figure 3:**
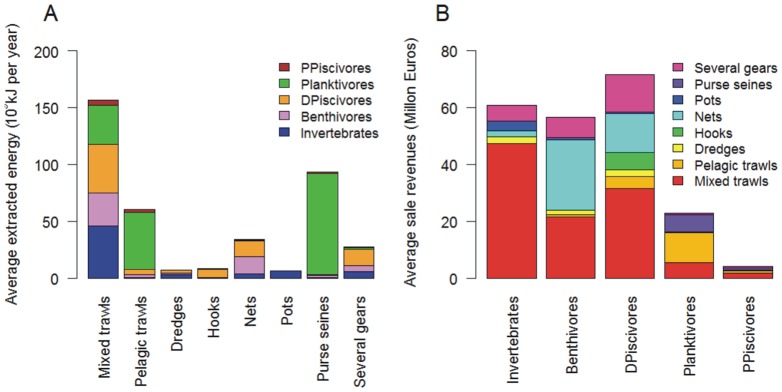
Average annual extracted energy (kJ) by fleet and functional group (A) and average annual sales revenues (€) derived from functional groups by each fleet during the period 2001–2009 (B). PPiscivores: pelagic piscivores; DPiscivores: demersal piscivores.

Mixed trawlers extracted on average 160 10^9^ kJ per year, corresponding to at least 50% of the energy contained in landings for all functional groups except planktivores for which the bulk of energy was extracted by purse seines, which landed around 89 10^9^ kJ of planktivores per year (Figure 3a). Pelagic trawlers contributed 61 10^9^ kJ of landings per year, corresponding to around 30% of extracted planktivore and pelagic piscivore energy. Vessel using trammel nets, driftnets or set gillnets, referred to as netters, contributed to the landings of all groups except planktivores. Unsurprisingly, pots were only used for benthic invertebrates while hooks contributed primarily to demersal piscivore landings.

As for the revenues fleets derived from functional groups (dependence), the fleets using hooks or pots were the most specialized as they drew more than 90% of their revenue from a single functional group, or four species (Table 1). The individual functional groups were demersal piscivores for hooks and benthic invertebrates for pots (Figure 3b). The average total annual revenues from invertebrates, benthivores and demersal piscivores were about equal, each functional group being worth around 60 millions € per year. These groups contributed primarily to the total revenues of mixed trawlers, netters, those using several gears or dredges and other passive gears. From the pelagic food web branch, pelagic planktivores were primarily contributing to the revenues of pelagic trawls and purse seines (Figure 3b).

Figure 1 shows the major contributions (>10%) of individual functional groups to the revenues and amounts of energy extracted by individual fleets. Four fleets depended on each functional group either in terms of revenue or landed energy or both, with the exception of demersal piscivores and planktivores for which it was six and three fleets respectively.

### Fleet returns

**Figure 4:**
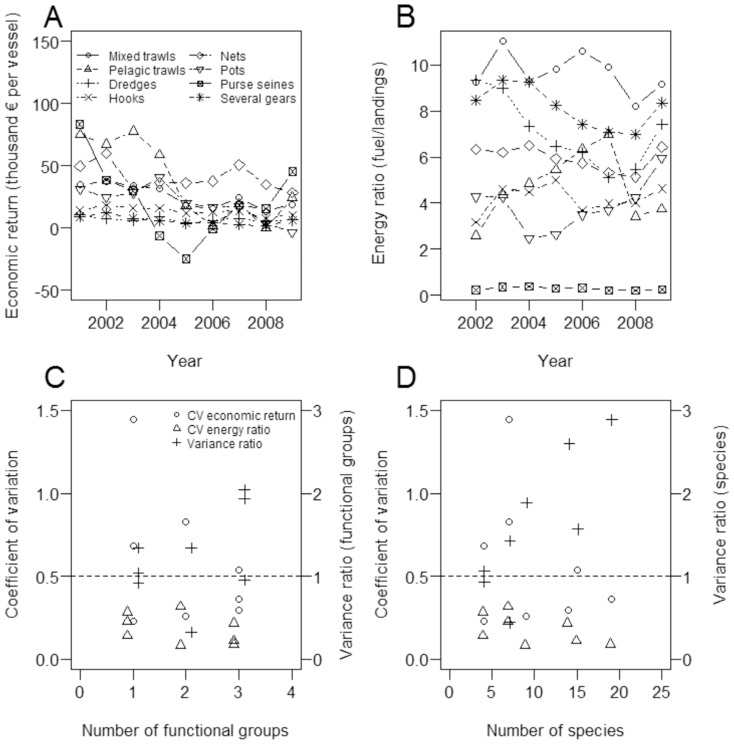
Economic returns (A), energy ratios (B), interannual coefficient of variation of economic returns and energy ratios, and variance ratios by fleet as a function of the number of functional groups exploited (C) and the number of species exploited (D). Variance ratio is interannual variance of mean vessel sales divided by the sum of interannual variances of species/functional group vessel sales for a given fleet.

The estimated economic return of most fleets decreased somewhat over the study period (Figure 4a). Nets and pelagic trawlers had the highest average annual economic return per vessel of 40,600 € and 38,100 € respectively (Table 1). However the estimated economic return of purse seiners was negative in 2004 and 2005 during the anchovy (*Engraulis encrasicolus*) low stock biomass and subsequent fishery closure in 2005. The lowest mean annual economic return per vessel was achieved by potters (2,000 € per vessel) and the fleet using several gears.

The average estimated ratio of fuel energy used to the energy content in the landings (live weight) ranged from 0.3 for purse seines to 9.7 for mixed trawls (Figure 4b, Table 1). The energy ratio tended to decrease while fuel price increased over 2003–2008, except for potters for which it increased. The ratio increased for all fleets in 2009, possibly because of a decrease in fuel price.

**Figure 5:**
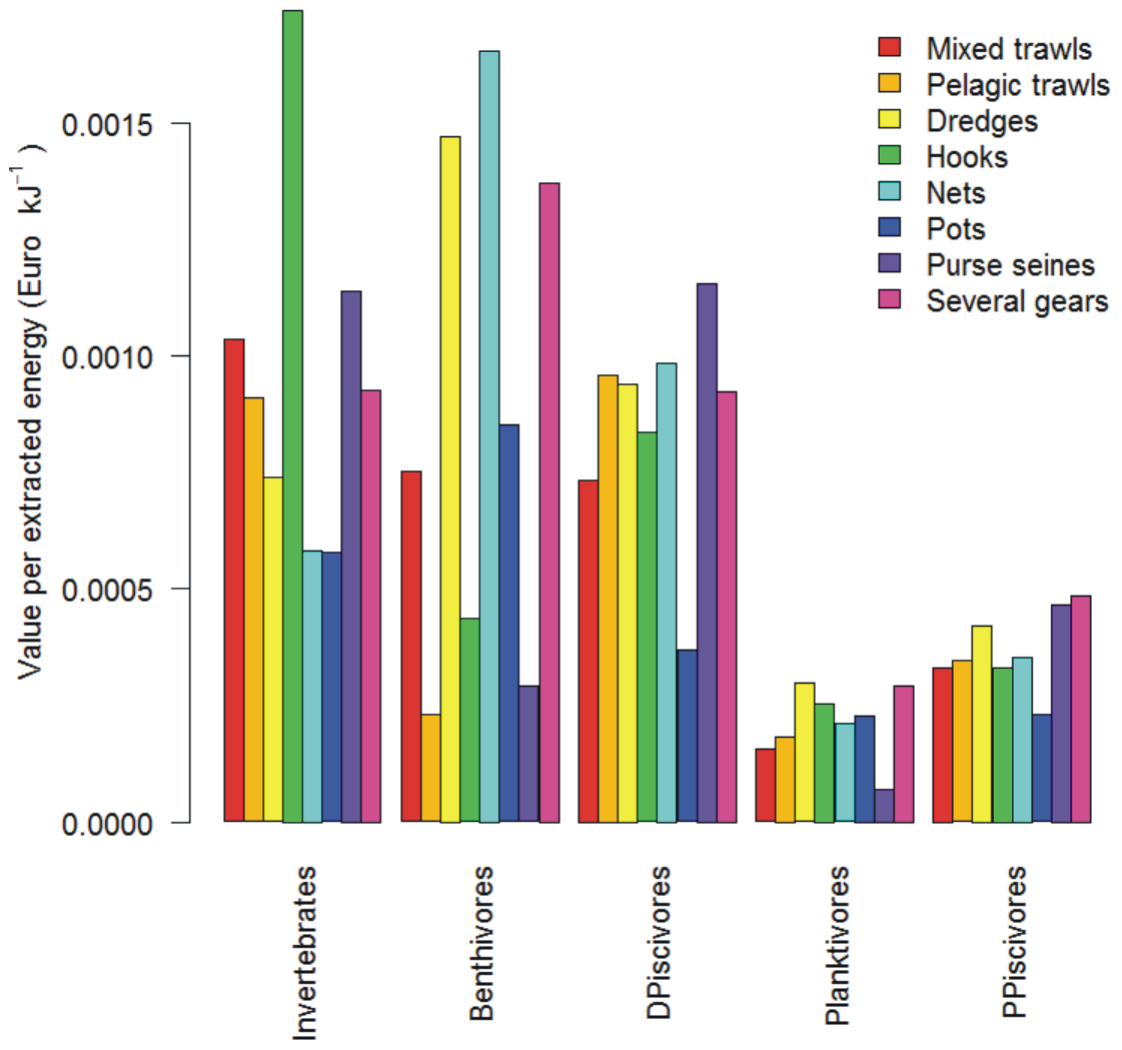
Average value-per-extracted-energy (€ kJ^−1^) for each functional group for French fleets fishing in the Bay of Biscay during the period 2001–2009.

The highest sale values-per-energy-extracted were achieved for the three functional groups of the demersal food web branch (Figure 5). However, large differences between fleets were observed; the differences were generally smaller for the pelagic groups. For example, for demersal benthivores the value-per-energy-extracted ranged from 0.23 € J^−1^ for pelagic trawls to 1.6 € J^−1^ for nets. For pelagic planktivores the range was 0.07 € J^−1^ for purse seines to 0.3 € J^−1^ for vessels using dredges and other active gears.

### Testing the portfolio theory

The ratio between the interannual variance of annual total revenues and the sum of interannual variances of functional group revenues was larger than one for four out of eight fleets (Table 1, Figure 4c), contradicting portfolio theory for these fleets. When using species for describing portfolio width, the ratio was larger than one for all but two fleets (Table 1, Figure 4d). The two exceptions were the vessels using hooks which primarily exploited four species and one functional group and pelagic trawls which targeted seven species in two functional groups (Table 1). Taking all fleets together neither the number of functional groups (p-value = 0.86) nor the number of species exploited (p-value = 0.99) were negatively correlated with the respective variance ratios (Figure 4c & d). On the contrary, variance ratios increased with the number of species.

Next, the interannual variability (coefficient of variation) of economic returns and energy ratios were each correlated with the number of species and the number of functional groups (Figure 4c & d). The interannual variability ranged from 0.23 to 1.44 for economic returns and from 0.09 to 0.32 for energy ratios. Economic return variability was neither related to the number of exploited species (p-value = 0.41) nor the number of exploited functional groups (p-value = 0.33). Thus, again these results do not support the predictions of portfolio theory for economic returns and energy ratios. The only result in support of portfolio theory was a weak stabilizing effect of catch diversity on energy ratio. The interannual variability for energy ratios decreased somewhat with the number of exploited species (Spearman's rho = −0.59, p-value = 0.06) but not with the number of exploited functional groups (Spearman's rho = −0.44, p-value = 0.12).

**Figure 6:**
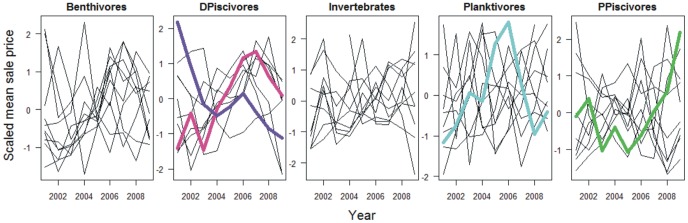
Time series of average scaled mean sale price for first ten species in each functional group for landings from the Bay of Biscay. Sea bass (pink), conger eel (purple), anchovy (blue) and albacore (green).

Finally, the average annual ex-vessel fish prices of the ten most important species of each functional group showed similar time trends for benthivores and demersal piscivores which would counteract a portfolio effect; prices were more independent for pelagic planktivores (Figure 6).

In summary, the results of this study indicate no reduction in interannual economic return variability with increasing portfolio width. The differences in interannual variability found between fleets point towards the importance of the species and functional groups that make up the portfolios.

## Discussion

The analysis of interannual variability in total revenues and economic returns of French fleets fishing in the Bay of Biscay did not generally reveal the compensations predicted by portfolio theory contrary to Chesapeake Bay [30], artisanal fishers in Dominica [31] or freshwater fisheries exploiting inland lakes [32]. In the Bay of Biscay, portfolio total revenues varied more than those for individual species for six out of eight fleets, the exceptions being pelagic trawls and the fleet using hooks. For pelagic trawlers, their target species can explain the findings. They exploited both the highly variable anchovy, whose fishery was closed for some years during the study period, and less variable but highly valuable species such as the sea bass and albacore [10], which in addition showed opposite time trends in ex-vessel prices compared to anchovy (Figure 6). The main target species for vessel using hooks were conger eel and sea bass [10], which also exhibited opposite time trends in ex-vessel prices (Figure 6).

For the remaining six fleets there are several possible explanations for the weak support of portfolio theory (see introduction): 1) synchronized abundance variations, 2) positively correlated ex-vessel prices and 3) non-selective fishing gears. We consider these three explanations in turn.

Synchrony in species abundances might prevent fishers to adjust their catches across species to stabilize their revenues. Indeed, trawl-survey based species abundance estimates of demersal piscivores in the Bay of Biscay were found to vary in synchrony [14]. In contrast, pelagic planktivore species showed signs of compensation (negative correlation) in the same study while benthivores had independent dynamics. Thus synchronized abundance variations could explain the findings for vessels using several gears as they drew the largest part of their revenues from demersal piscivores and provide part of the explanation for four other fleets except purse seiners.

Positively correlated ex-vessel fish prices are expected to lead to periods of generally high or low prices, the so called Law of One Price, which seems to operate at the European level e.g. Nielsen et al. [33]. Several species among benthivores, demersal piscivores and invertebrates showed periods of positive correlations of ex-vessel prices in the Bay of Biscay (Figure 6). These functional groups were the main contributors to the revenues of five fleets with little evidence of a portfolio effect the exception being again purse seiners. A more advanced analysis which is beyond this study is needed to investigate the detailed situation in the Bay of Biscay.

Unselective fishing gears might reduce the possibility to modify species targeting to compensate natural abundance fluctuations. Among the fleets with no clear portfolio effects, those using mixed trawls, dredges (combined with other gears) and several gears landed the largest number of species (14–19). Since the three fleets actually used several gears, they could modify their frequency of use to adjust the targeted species mix. Hence the type of fishing gear used does not seem to provide a plausible explanation for these three fleets.

There might be other explanations for the findings of this study. In longstanding fishing areas such as the Bay of Biscay all persisting vessels and fleets could have achieved a trade-off between economic return of invested capital and variability of return, independent of portfolio width. Thus, in the extreme situation, when an exploited population collapses vessels whose revenues strongly depended upon it simply disappear from the fleet and thus from the data set analysed. This may have occurred several times in the Bay of Biscay during the study period. In recent years, closures of fisheries for anchovy and porbeagle (*Lamna nasus*) gave rise to decommissioning plans but also vessels changing gears, or leaving the Bay of Biscay to fish elsewhere in the anchovy case [10], [34]. Thus, vessels with narrower portfolios or adaptability may have been decommissioned.

Finally, the benefits of a portfolio strategy might appear more strongly at the intra-annual, or even trip level instead of the interannual level investigated here. This would be the case if larger landings fetched lower prices per kg. Such a negative correlation between quantity landed and ex-vessel fish price was found for some species on the Brazilian market [9]. In this case it is beneficial for a vessel to land a range of species. Of course this effect depends on the type of fishery and target species.

Though we did not find any evidence in support of the portfolio theory, we found a large variability in economic and energy performance among fleets, but also years. The fleet difference could partly be due to different prices fetched for the same species by different fleets. A gear type and size effect was found for hake sold on the Spanish market [9]. On the Spanish market there were also large differences between prices of different size categories within fleets. Further, in our study vessel size varied between fleets. Most vessels using dredges, hooks, nets or pots were small (<12 m), while the trawler fleets contained generally larger vessels [25]. So vessel size might also explain part of the variability.

In this study energy ratios (fuel/landings) varied by a factor of thirty between fleets (average 0.3–9.7). Tyedmers et al. [35] in a study covering 250 distinct fisheries world wide found a global average of 12.5. This somewhat higher ratio than the energy ratios of French fleets in the Bay of Biscay may be explained by the fact that Tyedmers considered the proportion of energy contained in the edible part of the landings (muscle part of animal, typically 45–60% of total wet weight for fish [36]), whereas total wet weight was used here. However, it may probably still indicate that the French fleets were operating at relatively high energy ratios, i.e. high fuel consumption, in the global context, which subsidies (fuel aids) may have favoured [15]. The energy ratio estimates obtained here might aid the development of fuel efficient fishing methods for the Bay of Biscay as fuel efficient fishing is one of the current challenges faced by fisheries worldwide [37].

Pelagic planktivores contributed the largest share to energy landed from the Bay of Biscay food web. The picture is different if total revenues are considered. Benthic invertebrates, demersal benthivores and demersal piscivores contributed about the same to sale values, while the contribution of the two pelagic functional groups was smaller. This resulted in landed energy extracted from the demersal branch of the food web being worth more than that extracted from the pelagic branch. Thus demersal species had a higher value-for-energy-extracted. Note that this comparison excludes fishing costs which can be estimated at the fleet level, but are difficult to apportion to species or functional groups. Unfortunately no studies from other ecosystems were found to compare with these figures.

The unavailability of fishing cost data is often a hindrance in fisheries economic studies and leads to the use of values from other (hopefully similar) fisheries, e.g. Cheung and Sumaila [38]. Here we used estimates of fishing costs for all vessels in combination with registered landing values for calculating economic returns. This was possible as all explanatory variables of the cost models were available for all vessels, not only those included in the economic data sample. It is of course difficult to validate the estimates, but they are assumed to be reliable as the explained deviance of the different cost models were satisfactory (83–98%, Daurès et al. [25]). The cost and energy estimates could now also be used for developing and evaluating management options, carrying out management trade-off simulations or as input to value chain modelling [40]. In value chain calculations for a given food commodity not only the production, i.e. fishing costs are considered as we did here, but also the costs for vessel building, fish transformation, distribution, etc. Value chain modelling for the Bay of Biscay fisheries could identify the most cost-efficient or fuel-efficient fishing methods.

Data collected in fish auctions were used to estimate landings in volume and value. However, not all landings get sold in auctions hauls, so for certain vessels this lead to an underestimate of revenues. Based on the economic data sample it was estimated that around 90% of vessels sold at least part of their catch in a fish auction [25]. The vessels selling their catch directly and entirely outside the fish auction system were primarily small vessels (<12 m) using pots and other passive gears. The transformation of landings from weight into energy also lead to uncertainty as constant energy values per kg were used, ignoring size, sex, and seasonal differences; due to lack of data for certain species guess estimates had to be employed. This source of error should however have affected all fleets and functional groups in a similar manner.

There are many ways to group fishing vessels [39]. Here fleets were defined by a single characteristic, the dominant gears used in a given year. In previous studies of the French Bay of Biscay fishing vessels, fleets were defined using both a detailed list of gear combinations and the major fishing areas (coastal or shelf area) [10], [16]. Ignoring the fishing area in this study meant treating the Bay of Biscay food web as a whole, without distinguishing where energy was extracted, although we know that certain gears such as mixed trawlers included vessels fishing inshore or offshore or both [25]. Taking account of the distance to fishing grounds would primarily affect fuel costs thus possibly increase fleet economic returns for certain fleets. Alternatively, vessel size could have been taken into account. In the Bay of Biscay there is a strong link between fishing area, gears and vessel size [10].

In conclusion, little evidence was found in support of portfolio theory. Species composition rather than portfolio width seems to better explain the interannual variability of economic return of French fleets in the Bay of Biscay in recent years. As such, the predictions of portfolio theory do not seem to apply to the Bay of Biscay fisheries.

## Supporting Information

Table S1 **Functional group membership and energetic content.**
(DOC)
