# Supplementary material for: Interannual Variability of Fisheries Economic Returns and Energy Ratios Is Mostly Explained by Gear Type
Source: PLoS One. 2013 Jul 29;8(7):e70165. doi: 10.1371/journal.pone.0070165 (PMC3726391; doi:10.1371/journal.pone.0070165)
Supplement: Table S1 — Functional group membership and energetic content. (DOC) [file pone.0070165.s001.doc]

**Electronic supplementary material**

Trenkel et al. Portfolio composition not width reduces annual variability of fisheries economic returns and energy ratios.

**Table S1. Functional group membership and energetic content**.

NA not available. The "scaling method" consists of scaling the energy content *E* (kilo Calories) of a fillet found in Dorosz (1992) to whole fish live weight (kilo Joules) by multiplying with 6.7**E*.

| **English name** | **Latin name** | **Functional group** | **Energy (kJ per kg live weight)** | **Data source/Method** |
| --- | --- | --- | --- | --- |
| Small sandeel | *Ammodytes tobianus* | Benthivore | 5800 | Spitz et al 2010 |
| Imperial scaldfish | *Arnoglossus imperialis* | Benthivore | 5400 | Spitz et al 2010 |
| Red gurnard | *Aspitrigla cuculus* | Benthivore | 8200 | Spitz et al 2010 |
| Triggerfishes, durgons nei | *Balistidae* | Benthivore | 6900 | *Spondyliosoma cantharus* Spitz et al 2010 |
| Bogue | *Boops boops* | Benthivore | 8000 | Spitz et al 2010 |
| Dragonet | *Callionymus lyra* | Benthivore | 5200 | Spitz et al 2010 |
| Boarfish | *Capros aper* | Benthivore | 6200 | Spitz et al 2010 |
| Red band fish | *Cepola macrophthalma* | Benthivore | 3900 | Spitz et al 2010 |
| Streaked gurnard | *Chelidonichthys lastoviza* | Benthivore | 8200 | *Chelidonichthys cuculus* Spitz et al 2010 |
| Longfin gurnard | *Chelidonichthys obscurus* | Benthivore | 8200 | *Chelidonichthys cuculus* Spitz et al 2010 |
| Wedge sole | *Dicologlossa cuneata* | Benthivore | 6500 | Spitz et al 2010 |
| Grey gurnard | *Eutrigla gurnardus* | Benthivore | 8200 | *Chelidonichthys cuculus* Spitz et al 2010 |
| Silvery pout | *Gadiculus argenteus* | Benthivore | 5000 | Spitz et al 2010 |
| Witch flounder | *Glyptocephalus cynoglossus* | Benthivore | 5600 | Spitz et al 2010 |
| Blackbelly rose fish | *Helicolenus dactylopterus* | Benthivore | 9200 | Spitz et al 2010 |
| Amer. plaice(=Long rough dab) | *Hippoglossoides platessoides* | Benthivore | 6100 | *Lepidorhombus whiffiagonis* Spitz et al 2010 |
| Ballan wrasse | *Labrus bergylta* | Benthivore | 5400 | Spitz et al 2010 |
| Common dab | *Limanda limanda* | Benthivore | 5800 | *Pleuronectes platessa* Spitz et al. 2010 |
| Golden grey mullet | *Liza aurata* | Benthivore | 6500 | *Liza ramada* Spitz et al. 2010 |
| Thinlip grey mullet | *Liza ramada* | Benthivore | 6500 | *Liza ramada* Spitz et al. 2010 |
| Leaping mullet | *Liza saliens* | Benthivore | 6500 | *Liza ramada* Spitz et al. 2010 |
| Haddock | *Melanogrammus aeglefinus* | Benthivore | 3900 | *Merlangius merlangus* Spitz et al. 2010 |
| Thickback sole | *Microchirus variegatus* | Benthivore | 5000 | *Solea solea* Spitz et al 2010 |
| Lemon sole | *Microstomus kitt* | Benthivore | 5800 | Spitz et al 2010 |
| Flathead grey mullet | *Mugil cephalus* | Benthivore | 6500 | *Liza ramada* Spitz et al. 2010 |
| Red mullet | *Mullus surmuletus* | Benthivore | 6400 | Spitz et al 2010 |
| Axillary seabream | *Pagellus acarne* | Benthivore | 9400 | Spitz et al 2010 |
| Common pandora | *Pagellus erythrinus* | Benthivore | 9400 | *Pagellus acarne* Spitz et al. 2010 |
| Sand sole | *Pegusa lascaris* | Benthivore | 5000 | *Solea solea* Spitz et al 2010 |
| Greater forkbeard | *Phycis blennoides* | Benthivore | 5000 | Spitz et al 2010 |
| Flatfishes nei | *Pleuronectiformes* | Benthivore | 5700 | Pleuronectiformes Spitz et al. 2010 |
| Salema | *Sarpa salpa* | Benthivore | 7900 | *Scomber scombrus* Spitz et al. 2010 |
| Small-spotted catshark | *Scyliorhinus canicula* | Benthivore | 6400 | Spitz et al 2010 |
| Senegalese sole | *Solea senegalensis* | Benthivore | 5000 | *Solea solea* Spitz et al 2010 |
| Common sole | *Solea solea* | Benthivore | 5000 | Spitz et al 2010 |
| Black seabream | *Spondyliosoma cantharus* | Benthivore | 6900 | Spitz et al 2010 |
| Weevers nei | *Trachinus* | Benthivore | 5300 | Spitz et al 2010 |
| Greater weever | *Trachinus draco* | Benthivore | 5300 | Spitz et al 2010 |
| Mis. gurnards | *Trigla* | Benthivore | 8200 | *Chelidonichthys cuculus* Spitz et al 2010 |
| Piper gurnard | *Trigla lyra* | Benthivore | 8200 | *Chelidonichthys cuculus* Spitz et al 2010 |
| Gurnards, searobins nei | *Triglidae* | Benthivore | 8200 | *Chelidonichthys cuculus* Spitz et al 2010 |
| Pouting(=Bib) | *Trisopterus luscus* | Benthivore | 4700 | Spitz et al 2010 |
| European eel | *Anguilla anguilla* | DemPiscivore | 13700 | Scaling method |
| Black scabbardfish | *Aphanopus carbo* | DemPiscivore | 7900 | *Scomber scombrus* Spitz et al. 2010 |
| NA | *Argyrosomus* | DemPiscivore | 6000 | *Dicentrarchus labrax* Spitz et al 2010 |
| Meagre | *Argyrosomus regius* | DemPiscivore | 6000 | *Dicentrarchus labrax* Spitz et al 2010 |
| NA | *Beryx* | DemPiscivore | 9400 | *Pagellus acarne* Spitz et al. 2010 |
| European conger | *Conger conger* | DemPiscivore | 6900 | Spitz et al 2010 |
| Kitefin shark | *Dalatias licha* | DemPiscivore | 6400 | *Scyliorhinus canicula* Spitz et al 2010 |
| Common stingray | *Dasyatis pastinaca* | DemPiscivore | 5700 | *Leucoraja naevus* Spitz et al 2010 |
| European sea-bass | *Dicentrarchus labrax* | DemPiscivore | 6000 | Spitz et al 2010 |
| Spotted sea bass | *Dicentrarchus punctatus* | DemPiscivore | 6000 | *Dicentrarchus labrax* Spitz et al 2010 |
| Blue skate | *Dipturus batis* | DemPiscivore | 5700 | *Leucoraja naevus* Spitz et al. 2010 |
| Long nose skate | *Dipturus oxyrinchus* | DemPiscivore | 5700 | *Leucoraja naevus* Spitz et al. 2010 |
| Sharks, rays, skates, etc. nei | *Elasmobranchii* | DemPiscivore | 6000 | mean *Scyliorhinus canicula* & *Leucoraja naevus* Spitz et al 2010 |
| Dusky grouper | *Epinephelus marginatus* | DemPiscivore | 6000 | *Dicentrarchus labrax* Spitz et al 2010 |
| Gadiformes nei | *Gadiformes* | DemPiscivore | 3900 | *Merlangius merlangus* Spitz et al 2010 |
| Atlantic cod | *Gadus morhua* | DemPiscivore | 5300 | Scaling method |
| NA | *Galeorhinus* | DemPiscivore | 6400 | *Scyliorhinus canicula* Spitz et al 2010 |
| Tope shark | *Galeorhinus galeus* | DemPiscivore | 6400 | *Scyliorhinus canicula* Spitz et al 2010 |
| NA | *Hexanchus* | DemPiscivore | 6400 | *Scyliorhinus canicula* Spitz et al 2010 |
| Bluntnose sixgill shark | *Hexanchus griseus* | DemPiscivore | 6400 | *Scyliorhinus canicula* Spitz et al 2010 |
| NA | *Hippoglossus* | DemPiscivore | 6100 | *Lepidorhombus whiffiagonis* Spitz et al 2010 |
| Atlantic halibut | *Hippoglossus hippoglossus* | DemPiscivore | 6100 | *Lepidorhombus whiffiagonis* Spitz et al 2010 |
| Ratfishes nei | *Hydrolagus* | DemPiscivore | 3900 | *Chimaera monstrosa* Spitz et al. 2010 |
| NA | *Lepidopus* | DemPiscivore | 7900 | *Scomber scombrus* Spitz et al. 2010 |
| Silver scabbardfish | *Lepidopus caudatus* | DemPiscivore | 7900 | *Scomber scombrus* Spitz et al. 2010 |
| Megrim | *Lepidorhombus whiffiagonis* | DemPiscivore | 6100 | Spitz et al 2010 |
| Sandy ray | *Leucoraja circularis* | DemPiscivore | 5700 | *Leucoraja naevus* Spitz et al 2010 |
| Shagreen ray | *Leucoraja fullonica* | DemPiscivore | 5700 | *Leucoraja naevus* Spitz et al. 2010 |
| Cuckoo ray | *Leucoraja naevus* | DemPiscivore | 5700 | Spitz et al 2010 |
| Anglerfish nei | *Lophius* | DemPiscivore | 3700 | *Merluccius merluccius* Spitz et al. 2010 |
| Blackbellied angler | *Lophius budegassa* | DemPiscivore | 3700 | *Merluccius merluccius* Spitz et al. 2010 |
| Monk | *Lophius piscatorius* | DemPiscivore | 3700 | *Merluccius merluccius* Spitz et al. 2010 |
| Whiting | *Merlangius merlangus* | DemPiscivore | 3900 | Spitz et al 2010 |
| European hake | *Merluccius merluccius* | DemPiscivore | 3700 | Spitz et al 2010 |
| Spanish ling | *Molva macrophthalma* | DemPiscivore | 3900 | *Merlangius merlangus* Spitz et al. 2010 |
| Ling | *Molva molva* | DemPiscivore | 4000 | *Pollachius pollachius* Spitz et al 2010 |
| Smoothhound | *Mustelus* | DemPiscivore | 6000 | *Scyliorhinus canicula* Spitz et al 2010 |
| Common eagle ray | *Myliobatis aquila* | DemPiscivore | 5700 | *Leucoraja naevus* Spitz et al. 2010 |
| Blackspot seabream | *Pagellus bogaraveo* | DemPiscivore | 9400 | *Pagellus acarne* Spitz et al. 2010 |
| Red porgy | *Pagrus pagrus* | DemPiscivore | 6900 | *Spondyliosma cantharus* Spitz et al. 2010 |
| European flounder | *Platichthys flesus* | DemPiscivore | 5200 | Scaling method |
| European plaice | *Pleuronectes platessa* | DemPiscivore | 5800 | Spitz et al 2010 |
| Pollack | *Pollachius pollachius* | DemPiscivore | 4200 | Spitz et al 2010 |
| Saithe(=Pollock) | *Pollachius virens* | DemPiscivore | 4200 | *Pollachius pollachius* Spitz et al. 2010 |
| Wreckfish | *Polyprion americanus* | DemPiscivore | 6000 | *Dicentrarchus labrax* Spitz et al 2010 |
| Turbot | *Psetta maxima* | DemPiscivore | 6000 | Solidae Spitz et al 2010 |
| Thornback ray | *Raja clavata* | DemPiscivore | 5700 | *Leucoraja naevus* Spitz et al 2010 |
| Small-eyed ray | *Raja microocellata* | DemPiscivore | 5700 | *Leucoraja naevus* Spitz et al. 2010 |
| Spotted ray | *Raja montagui* | DemPiscivore | 5700 | *Leucoraja naevus* Spitz et al 2010 |
| Undulate ray | *Raja undulata* | DemPiscivore | 5700 | *Leucoraja naevus* Spitz et al. 2010 |
| Rays and skates nei | *Rajidae* | DemPiscivore | 5700 | *Leucoraja naevus* Spitz et al 2010 |
| Brill | *Scophthalmus rhombus* | DemPiscivore | 6000 | Solidae Spitz et al 2010 |
| Black scorpionfish | *Scorpaena porcus* | DemPiscivore | 5100 | Scorpaenidae Spitz et al. 2010 |
| Red scorpionfish | *Scorpaena scrofa* | DemPiscivore | 5100 | Scorpaenidae Spitz et al. 2010 |
| Nursehound | *Scyliorhinus stellaris* | DemPiscivore | 6400 | *Scyliorhinus canicula* Spitz et al 2010 |
| Atlantic redfish | *Sebastes* | DemPiscivore | 9200 | *Helicolenus dactylopterus* Spitz et al. 2010 |
| Gilthead seabream | *Sparus aurata* | DemPiscivore | 8000 | Sparidae Spitz et al 2010 |
| Dogfish sharks nei | *Squalidae* | DemPiscivore | 6400 | *Scyliorhinus canicula* Spitz et al 2010 |
| Picked dogfish | *Squalus acanthias* | DemPiscivore | 6400 | *Scyliorhinus canicula* Spitz et al 2010 |
| Angelshark | *Squatina squatina* | DemPiscivore | 6400 | *Scyliorhinus canicula* Spitz et al 2010 |
| Marbled electric ray | *Torpedo marmorata* | DemPiscivore | 5700 | *Lecoraja naevus* Spitz et al 2010 |
| NA | *Trachyscorpia* | DemPiscivore | 9200 | *Helicolenus dactylopterus* Spitz et al. 2010 |
| Drums nei | *Umbrina* | DemPiscivore | 6000 | *Dicentrarchus labrax* Spitz et al 2010 |
| Canary drum (=Baardman) | *Umbrina canariensis* | DemPiscivore | 6000 | *Dicentrarchus labrax* Spitz et al 2010 |
| John dory | *Zeus faber* | DemPiscivore | 9400 | *Pagellus acarne* Spitz et al 2010 |
| Mis. clams, cockles | *Bivalvia* | Invertebrates | 3100 | Scaling method |
| NA | *Callista* | Invertebrates | 3100 | Scaling method |
| Smooth callista | *Callista chione* | Invertebrates | 3100 | Scaling method |
| Edible crab | *Cancer pagurus* | Invertebrates | 5600 | Decapoda brachyurans Spitz et al. 2010 |
| Green crab | *Carcinus maenas* | Invertebrates | 6900 | *Necora puber* Spitz et al. 2010 |
| Cephalopods nei | *Cephalopoda* | Invertebrates | 3600 | *Sepia officinalis* Spitz et al. 2010 |
| Cockles | *Cerastoderma* | Invertebrates | 3100 | Scaling method |
| Common edible cockle | *Cerastoderma edule* | Invertebrates | 3100 | Scaling method |
| Crangon shrimps nei | *Crangon* | Invertebrates | 3400 | *Palaemon longirostris* Spitz et al 2010 |
| Common shrimp | *Crangon crangon* | Invertebrates | 3400 | *Palaemon longirostris* Spitz et al 2010 |
| Pacific cupped oyster | *Crassostrea gigas* | Invertebrates | 5300 | Scaling method |
| Horned and musky octopuses | *Eledone* | Invertebrates | 4700 | Spitz et al 2010 |
| Horned octopus | *Eledone cirrhosa* | Invertebrates | 4700 | Spitz et al 2010 |
| Craylets, squat lobsters | *Galatheidae* | Invertebrates | 6900 | *Necora puber* Spitz et al 2010 |
| Dog cockle | *Glycymeris glycymeris* | Invertebrates | 3100 | Scaling method |
| European lobster | *Homarus gammarus* | Invertebrates | 5700 | Scaling method |
| Inshore squids nei | *Loliginidae* | Invertebrates | 4000 | Spitz et al 2010 |
| NA | *Maja* | Invertebrates | 5600 | Decapoda brachyurans Spitz et al. 2010 |
| Spinous spider crab | *Maja squinado* | Invertebrates | 5600 | Decapoda brachyurans Spitz et al. 2010 |
| European mussel | *Mytilus* | Invertebrates | 7900 | Scaling method |
| Velvet swimcrab | *Necora puber* | Invertebrates | 6900 | Spitz et al .2010 |
| Norway lobster | *Nephrops norvegicus* | Invertebrates | 6900 | *Necora puber* Spitz et al. 2010 |
| Octopuses nei | *Octopus* | Invertebrates | 4700 | *Eledone cirrhosa* Spitz et al. 2010 |
| Common octopus | *Octopus vulgaris* | Invertebrates | 4700 | *Eledone cirrhosa* Spitz et al. 2010 |
| European flat oyster | *Ostrea edulis* | Invertebrates | 5300 | Scaling method |
| Delta prawn | *Palaemon longirostris* | Invertebrates | 3400 | Spitz et al 2010 |
| Prawn | *Palaemon serratus* | Invertebrates | 3400 | *Palaemon longirostris* Spitz et al 2010 |
| Common spiny lobster | *Palinurus elephas* | Invertebrates | 5700 | Scaling method |
| Pink spiny lobster | *Palinurus mauritanicus* | Invertebrates | 6000 | Scaling method |
| Deepwater rose shrimp | *Parapenaeus longirostris* | Invertebrates | 3400 | *Palaemon longirostris* Spitz et al 2010 |
| Common scallop | *Pecten maximus* | Invertebrates | 5100 | Scaling method |
| Grooved carpet shell | *Ruditapes decussatus* | Invertebrates | 5300 | Scaling method |
| Japanese carpet shell | *Ruditapes philippinarum* | Invertebrates | 5300 | Scaling method |
| Elegant cuttlefish | *Sepia elegans* | Invertebrates | 3600 | *Sepia officinalis* Spitz et al. 2010 |
| Common cuttlefish | *Sepia officinalis* | Invertebrates | 3800 | Spitz et al 2010 |
| Pink cuttlefish | *Sepia orbignyana* | Invertebrates | 3600 | *Sepia officinalis* Spitz et al. 2010 |
| Surf clams nei | *Spisula* | Invertebrates | 3100 | Scaling method |
| Mis. tellin | *Tellina* | Invertebrates | 3100 | Scaling method |
| NA | *Todaropsis* | Invertebrates | 4400 | Spitz et al 2010 |
| Lesser flying squid | *Todaropsis eblanae* | Invertebrates | 4400 | Spitz et al 2010 |
| NA | *Venerupis* | Invertebrates | 3100 | Scaling method |
| NA | *Venus* | Invertebrates | 3500 | Scaling method |
| Warty venus | *Venus verrucosa* | Invertebrates | 3500 | Scaling method |
| Thresher | *Alopias vulpinus* | PelPiscivore | 7900 | *Scomber scombrus* Spitz et al. 2010 |
| NA | *Brama* | PelPiscivore | 6000 | Carangidae Spitz et al 2010 |
| Atlantic pomfret | *Brama brama* | PelPiscivore | 6000 | Carangidae Spitz et al 2010 |
| NA | *Katsuwonus* | PelPiscivore | 7900 | *Scomber scombrus* Spitz et al. 2010 |
| Skipjack tuna | *Katsuwonus pelamis* | PelPiscivore | 7900 | *Scomber scombrus* Spitz et al. 2010 |
| NA | *Lamna* | PelPiscivore | 7900 | *Scomber scombrus* Spitz et al. 2010 |
| Porbeagle | *Lamna nasus* | PelPiscivore | 7900 | *Scomber scombrus* Spitz et al. 2010 |
| NA | *Petromyzon* | PelPiscivore | 7900 | *Scomber scombrus* Spitz et al. 2010 |
| Sea lamprey | *Petromyzon marinus* | PelPiscivore | 7900 | *Scomber scombrus* Spitz et al. 2010 |
| NA | *Prionace* | PelPiscivore | 7900 | *Scomber scombrus* Spitz et al. 2010 |
| Blue shark | *Prionace glauca* | PelPiscivore | 7900 | *Scomber scombrus* Spitz et al. 2010 |
| Atlantic salmon | *Salmo salar* | PelPiscivore | 7200 | *Dicentrarchus labrax* + 20% Spitz et al 2010 |
| Atlantic bonito | *Sarda sarda* | PelPiscivore | 7900 | *Scomber scombrus* Spitz et al. 2010 |
| Atlantic white marlin | *Tetrapturus albidus* | PelPiscivore | 6200 | Perciformes Spitz et al. 2010 |
| Albacore | *Thunnus alalunga* | PelPiscivore | 7900 | *Scomber scombrus* Spitz et al. 2010 |
| Bigeye tuna | *Thunnus obesus* | PelPiscivore | 7900 | *Scomber scombrus* Spitz et al. 2010 |
| Atlantic bluefin tuna | *Thunnus thynnus* | PelPiscivore | 7900 | *Scomber scombrus* Spitz et al. 2010 |
| NA | *Xiphias* | PelPiscivore | 7900 | *Scomber scombrus* Spitz et al. 2010 |
| Swordfish | *Xiphias gladius* | PelPiscivore | 7900 | *Scomber scombrus* Spitz et al. 2010 |
| Veined squid | *Loligo forbesi* | Planktivore | 4600 | Spitz et al 2010 |
| European squid | *Loligo vulgaris* | Planktivore | 4800 | Spitz et al 2010 |
| Squids nei | *Ommastrephidae* | Planktivore | 4300 | cephalopodes Spitz et al 2010 |
| Twaite shad | *Alosa fallax* | Planktivore | 10700 | Scaling method |
| Greater atlantic argentine | *Argentina silus* | Planktivore | 6100 | *Argentina sphyraena* Spitz et al 2010 |
| Argentine | *Argentina sphyraena* | Planktivore | 6100 | Spitz et al 2010 |
| Garfish | *Belone belone* | Planktivore | 6200 | Spitz et al 2010 |
| NA | *Clupea* | Planktivore | 10200 | Spitz et al 2010 |
| Atlantic herring | *Clupea harengus* | Planktivore | 10200 | Spitz et al 2010 |
| anchovies | *Engraulis* | Planktivore | 5800 | Spitz et al 2010 |
| European anchovy | *Engraulis encrasicolus* | Planktivore | 5800 | Spitz et al 2010 |
| NA | *Micromesistius* | Planktivore | 4400 | Spitz et al 2010 |
| Blue whiting(=Poutassou) | *Micromesistius poutassou* | Planktivore | 4400 | Spitz et al 2010 |
| European smelt | *Osmerus eperlanus* | Planktivore | 5200 | Scaling method |
| NA | *Sardina* | Planktivore | 8700 | Spitz et al 2010 |
| European pilchard(=Sardine) | *Sardina pilchardus* | Planktivore | 8700 | Spitz et al 2010 |
| Atlantic chub mackerel | *Scomber colias* | Planktivore | 7900 | *Scomber scombrus* Spitz et al. 2010 |
| Chub mackerel | *Scomber japonicus* | Planktivore | 7900 | *Scomber scombrus* Spitz et al. 2010 |
| Atlantic mackerel | *Scomber scombrus* | Planktivore | 7900 | Spitz et al 2010 |
| Caribbean king mackerels | *Scomberesox* | Planktivore | 5800 | Spitz et al 2010 |
| European sprat | *Sprattus sprattus* | Planktivore | 6500 | Spitz et al 2010 |
| Mediterranean horse mackerel | *Trachurus mediterraneus* | Planktivore | 6000 | *Trachurus trachurus* Spitz et al. 2010 |
| Atlantic horse mackerel | *Trachurus trachurus* | Planktivore | 6000 | Spitz et al 2010 |

**References**

Dorosz Ph (1992) Table des calories.

Spitz J, Mourocq E, Schoen V, Ridoux V (2010) Proximate composition and energy content of forage species from the Bay of Biscay: high- or low-quality food? ICES Journal of Marine Science 67: 909-915.
